# Altering Opioid Neuromodulation in the Songbird Basal Ganglia Modulates Vocalizations

**DOI:** 10.3389/fnins.2019.00671

**Published:** 2019-07-03

**Authors:** Sandeep Kumar, Alok Nath Mohapatra, Hanuman Prasad Sharma, Utkarsha A. Singh, Niranjan Ashok Kambi, Thirumurthy Velpandian, Raghav Rajan, Soumya Iyengar

**Affiliations:** ^1^National Brain Research Centre, Gurgaon, India; ^2^Department of Ocular Pharmacology and Pharmacy, Dr. R. P. Centre, All India Institute of Medical Sciences, New Delhi, India; ^3^Indian Institute of Science Education and Research, Pune, Pune, India

**Keywords:** μ-opioid receptors, motivation, vocalization, songbirds, zebra finches, basal ganglia, dopamine

## Abstract

Although the interplay between endogenous opioids and dopamine (DA) in the basal ganglia (BG) is known to underlie diverse motor functions, few studies exist on their role in modulating speech and vocalization. Vocal impairment is a common symptom of Parkinson’s disease (PD), wherein DA depletion affects striosomes rich in μ-opioid receptors (μ-ORs). Symptoms of opioid addiction also include deficiencies in verbal functions and speech. To understand the interplay between the opioid system and BG in vocalization, we used adult male songbirds wherein high levels of μ-ORs are expressed in Area X, a BG region which is part of a circuit similar to the mammalian thalamocortical-basal ganglia loop. Changes in DA, glutamate and GABA levels were analyzed during the infusion of different doses of the μ-OR antagonist naloxone (50 and 100 ng/ml) specifically in Area X. Blocking μ-ORs in Area X with 100 ng/ml naloxone led to increased levels of DA in this region without altering the number of songs directed toward females (FD). Interestingly, this manipulation also led to changes in the spectro-temporal properties of FD songs, suggesting that altered opioid modulation in the thalamocortical-basal ganglia circuit can affect vocalization. Our study suggests that songbirds are excellent model systems to explore how the interplay between μ-ORs and DA modulation in the BG affects speech/vocalization.

## Highlights

- μ-ORs are expressed by medium spiny and pallidal output neurons of Area X, a basal ganglia homologue in songbirds.
- Blocking μ-ORs in Area X with 100 g/ml naloxone leads to changes in the acoustic properties of vocalization and an increase in DA levels in Area X.
- Despite the increase in DA levels within Area X, blocking μ-ORs did not lead to changes in the number of songs.

## Introduction

Previous studies demonstrated significant deficits in verbal intelligence (Prosser et al., 2006) and speech deficits (slurring and/or stuttering) in opioid addicts (Wasserman and Yahr, 1980) and as side-effects of prescription opioids. Further, social approach behaviors and the production of ultrasonic vocalizations were partially regulated by μ-opioid ligands in juvenile and adult male rodents (Wöhr and Schwarting, 2009; Buck et al., 2014). Taken together, these studies suggest that the opioid system modulates social aspects of vocal behavior and vocalization itself. Whereas most studies have explored the role of opioid modulation in reward-based learning or in motivated behaviors, its role in vocalization has not been explored thoroughly.

Songbirds such as zebra finches (*Taenopygia guttata*) which use their songs for courtship have been extensively used to study how vocalizations are learned during development and sung in different social contexts in adulthood. Both of these functions are controlled by the anterior forebrain pathway (AFP), a part of the song control system [SCS, (Nottebohm et al., 1976), Figure 1A]. The AFP is organized in the form of a thalamocortical-BG loop, akin to those in mammalian brains. Interestingly, different components of the SCS including AFP in male songbirds express high levels of ORs (opioid receptors) (Khurshid et al., 2009). Systemically blocking μ-ORs (μ-opioid receptors) with different doses of the opioid antagonist naloxone in adult male zebra finches led to decreases in the number of songs and alterations in their acoustic features (Khurshid et al., 2010).

**FIGURE 1:**
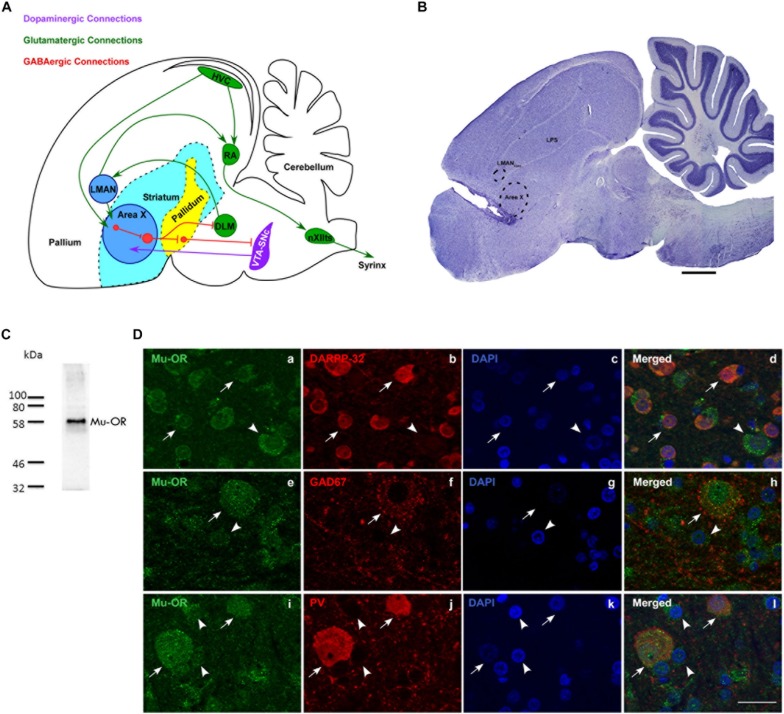
**(A)** Schematic of the song control system and its connections [adapted with permission from the Journal of Comparative Neurology through Copyright Clearance Center’s RightsLink^®^ service (Nottebohm et al., 1976; Farries et al., 2005; Gale et al., 2008)] showing components of the vocal motor pathway (VMP; HVC→ RA→ nXIIts) and the anterior forebrain pathway (AFP; LMAN→ Area X→ DLM→LMAN. Of these regions, HVC, RA, and LMAN are located in the avian pallium (cortex). The AFP connects to VMP via projections from LMAN to RA and connections from HVC to Area X. HVC, cortical premotor nucleus (abbreviation used as a formal name); RA, robust nucleus of the arcopallium; DLM, dorsolateral nucleus of medial thalamus; LMAN, lateral magnocellular nucleus of the anterior nidopallium; Area X, a nucleus of the avian basal ganglia; VTA-SNc, ventral tegmental area-substantia nigra complex; nXIIts, tracheosyringeal part of the hypoglossal nucleus; Glutamatergic, GABAergic and dopaminergic pathways are indicated by green, red, and purple arrows. **(B)** A sagittal Nissl-stained section showing the location of the probe in the rostral-most part of Area X. Dotted lines indicate the borders of Area X and LMAN_core_. Scale bar, 1 mm. **(C)** Validation for the specificity of the antibody used against μ-ORs (Abcam, ab10275, polyclonal). A single band was observed at the predicted molecular weight of ∼59–65 kDa. **(D)** Striatal (medium spiny) neurons in Area X (arrows) co-express μ-ORs (**a**; green) and DARPP-32 (**b**; red), nuclei labeled with DAPI (**c**; blue); merged image **(d)**. The arrowhead indicates a μ-OR-positive pallidal neuron which is not positive for DARPP-32. GAD67-positive and parvalbumin-positive neurons in Area X co-express μ-ORs. The arrow in **(e)** indicates the expression of μ-ORs (green) in Area X with GAD67 (red, **f**) expressed by a GPi neuron seen in the merged image **(h)**. A striatal neuron (arrowhead, **e**) with very low levels of GAD67 **(f,h)** can also be seen. The lower panel **(i)** shows two μ-OR-labeled neurons (green, arrows) which are also positive for parvalbumin (PV, red, **j**) seen in the merged image **(l)**. Smaller striatal neurons in this panel **(i–l)** are indicated by arrowheads and are not PV-positive, DAPI in all sets of figures in the third column **(c,g,k)** labels neuronal nuclei. Scale bar, 20 μm.

A major component of the AFP, that is, Area X (an avian BG nucleus) is composed of striatal and pallidal GABAergic neurons (Farries and Perkel, 2002), both of which express μ-ORs (inhibitory G-protein coupled receptors; Connor and Christie, 1999; Koehl et al., 2018) and enkephalin (Carrillo and Doupe, 2004) as seen in the mammalian basal ganglia. Area X contributes to vocal learning in juveniles (Sohrabji et al., 1990) and singing in different social contexts in adulthood (Jarvis et al., 1998) and is densely innervated by dopaminergic projections (Bottjer, 1993). Similarly, high levels of μ-ORs are expressed in the human BG, which is known to modulate motivation and emotion-driven changes in vocalization (reviewed by Ziegler and Ackermann, 2017). Interestingly, Sasaki et al. (2006) demonstrated an increase in DA levels in Area X during FD (female-directed) singing compared to those when males were silent or sang alone. However, Ihle et al. (2015) showed that changes in DA levels in Area X were not necessarily coupled with singing and were instead linked to heightened arousal and attentional states during courtship. Singing was followed by an increase in enkephalin expression in Area X and HVC (Wada et al., 2006), probably to decrease the excessive activation of striatal neurons by DA (Steiner and Gerfen, 1998). Further, context-dependent changes were reported in the spectral features of FD songs after DA modulation in Area X, but not in temporal features (Leblois and Perkel, 2012). Therefore, it remains poorly understood whether DA levels within Area X are modulated by the opioid system and what specific effects both neuromodulatory systems may have on vocalizations. Based on these parallels, we hypothesized that changes in vocalization might be modulated by endogenous opioids in the BG, which we decided to test in adult male zebra finches.

## Materials and Methods

All experimental procedures were approved by the Institutional Animal Ethics Committee at the National Brain Research Centre, Manesar, India in accordance with recommendations of the Committee for the Purpose of Control and Supervision of Experiments on Animals (CPCSEA), India. Adult male zebra finches (>120 days of age; *n* = 10) were used for the present study. Animals were given *ad libitum* access to food and water and maintained at a 12/12-h light and dark cycle.

### Anesthesia and Surgery

Birds were anesthetized using intramuscular (I.M.) injections of ketamine [25 mg/kg body weight (bw)], xylazine (2.5 mg/kg bw), and diazepam (8 mg/kg bw). The induction dose also contained the analgesic Butorphanol tartrate (Butrum, I.M., 0.1 mg/kg bw). A tungsten electrode (1 MΩ at 1 kHz, Microprobe Inc., MD, United States) was lowered into the brain through a burr hole drilled in the skull over the left hemisphere 6.5 mm rostral and 1.4 mm lateral to the bifurcation of the superior sagittal sinus at 38° to avoid penetrating the overlying lateral magnocellular nucleus of anterior nidopallium (LMAN). Increases in spontaneous activity in response to playbacks of the bird’s own songs (BOS) were used to confirm the boundaries of Area X (Figure 1B; Doupe, 1997). A guide cannula (CMA 7, Microdialysis, Solna, Sweden) was implanted into the brain at the anterior boundary of Area X at a depth of 2.2–2.3 mm to ensure minimal damage to Area X (Leblois and Perkel, 2012). It was fixed in place with cyanoacrylate glue and epoxy, after which birds were returned to their home cages. After surgery, birds were kept in their cages near a heat source to keep them warm and injected with 0.3 mg/kg bw of I.M. Flumazenil (F6300, Sigma-Aldrich) to counteract the effect of diazepam (Prather, 2012). In the evening following the surgery and for the next three days, birds were injected with the analgesic (Butrum 0.1 mg/kg bw, I.M.) and also provided with the oral antibiotic Cephalexin (35 mg/kg bw; Phexin Dry Syrup, GlaxoSmithKline Pharmaceuticals Limited).

### Microdialysis Setup

A syringe pump (CMA 402, CMA Microdialysis) connected to a microdialysis probe (CMA-7, Cuprophane membrane length 1 mm, CMA Microdialysis) was used to infuse artificial cerebrospinal fluid (aCSF, pH 6; composition: NaCl 147 mM/L, KCl 2.7 mM/L, CaCl_2_ 1.2 mM/L, MgCl_2_ 0.85 mM/L) or the drug (naloxone, N7758 Sigma) dissolved in aCSF. To allow the bird to move freely, the probe was attached to the guide cannula via a swivel (375/D/22 Dual channel swivel, 22ga, Instech Laboratories, Inc., Plymouth, United States) suspended from a Multi-Axis Counter-Balanced Lever Arm (SMCLA, 9 cm, Instech Laboratories, Inc., Plymouth, United States). The microdialysis probe was inserted into the guide cannula after flushing it with aCSF on the eve of the experiment. Next morning, before the actual experiments, aCSF was infused at the rate of 3 μl/min for 10–15 min to let the flow rate stabilize. During the experiments, dialysates were collected in the dark on ice in an Eppendorf tube containing 1 mM ascorbic acid (05878, Sigma-Aldrich, United States; 15% of the total dialysate volume) to minimize oxidative degradation of DA. Dialysate samples were lyophilized and stored at −80^∘^C until they were analyzed.

### Estimation of Neurotransmitters by LC-MS/MS

Levels of GABA (gamma-aminobutyric acid), glutamate and DA were analyzed in lyophilized dialysate samples from seven (out of ten) birds using LC-MS/MS (liquid chromatography-tandem mass spectrometry) adopted from González et al. (2011) with minor modifications. The Ultra High-Performance Liquid Chromatography (UHPLC, Accela, Thermo Surveyor system, Thermo Electron Corp., Waltham, MA, United States) was coupled with a triple quadrupole mass spectrometer (4000 Q-Trap, AB/MDS Sciex, Foster City, CA, United States) and operated in the positive electrospray ionization mode.

Chromatographic parameters were controlled by the Chromquest software (version 4.1). A cocktail solution of the three neurotransmitter standards [gamma aminobutyric acid (A2129); L-Glutamate, (G1251) and DA hydrochloride (PHR1090), 100 ng/ml; Sigma-Aldrich Pvt. Ltd., United States] dissolved in a solvent consisting of 0.1 M formic acid (FA) and 15% 1 mM ascorbic acid in milliQ water was loaded onto the Acquity UPLC BEH C18 column (Waters, India Pvt. Ltd.) to achieve separation. The mobile phase consisted of the eluent-A (methanol), and the second eluent-B [0.05% (v/v) FA in water with 1 mM of heptafluorobutyric acid]. The gradient run (8 min) was started with 5% of A (0 to 1st min, 0.2 ml/min), then shifted to 100% of A (1.5th to 5.5th min, 0.4 ml/min). The first line condition was achieved by the 6th min and maintained for the next 2 min. The column oven and sample tray were maintained at RT.

The Analyst software (version 1.5.2) was used to control mass parameters and data acquisition. The multiple reaction monitoring mode was used for the quantification of neurotransmitters. Whereas source-dependent parameters included curtain gas (CUR), 30 psi; collision gas (CAD), 6 psi; ion source voltage, 5.5 KV and source temperature at 400^∘^C; ion source gas (GS1 and GS2), at 40 and 60 psi, respectively, declustering (30V) and entrance potentials (10V) had dwell times of 100 ms. The following m/z transitions and collision energies were used for quantification and confirmation, respectively: GABA (104.0→87.0, 14V and 104.0→58.1, 25V), glutamate (148.0→130.0, 14V and 148.0→84.0, 22V) and DA (154.1→137, 10V and 154.1→119.0, 25V; 154.1→91.1, 30V).

### Behavioral Recordings

Experiments were performed in a custom-made sound-attenuated box 4–7 days after surgery when singing returned to baseline levels. All experiments were conducted in the morning starting between 8.30 and 9.00 am, within 1 h of switching on the lights and were concluded by ∼11.30 am. A set of 3–4 female birds were placed in a cage opposite experimental birds to elicit FD singing. Occasionally, if males did not sing, the first set of females was replaced by another. Dialysates were collected for 45 min during aCSF infusions and for 35 min during naloxone infusion [50 ng/ml (8/10 birds); 100 ng/ml (9/10 birds)], separated by 50 min of audio-visual isolation from the females. Each set of control and naloxone infusions were performed in the morning for at least 3–4 days. All audio recordings were obtained with a microphone (Sennheiser e614) connected to a computer via an M-Track Quad device (M-Audio) and the SA+ recorder (Sound Analysis Pro software version 1.02) (Tchernichovski et al., 2000). Male birds’ behavior was recorded with a webcam (Logitech HD C210). Only those FD motifs/songs were analyzed during which males sang near females (either facing them or sideways), which occurs in natural courtship.

### Western Blot

Immunoblotting was used to validate the specificity of the anti-μ-OR antibody (1:5000; Abcam, ab10275, polyclonal; made in rabbit, which detects a band of 49–65 kDa) and peroxidase-labeled secondary antibody (anti-rabbit; 1:6000, Vector Laboratories, PI-1000) used to detect μ-ORs in zebra finches (Figure 1C; cf. Sen et al., 2019). The anti-μ-OR antibody (ab10275) was raised against a sequence of 15 amino acids on the C-terminal (384 to 398) of OPRM-1; NX_P35372, which is a synthetic rat μ-OR peptide. This 15 amino acid sequence has 80% (12/15) homology to a similar sequence found in μ-ORs present in songbirds [zebra finch, (XP_012428092.1) and in starlings, (XP_014741492.1; (Kelm et al., 2011)]. Whereas the μ-OR sequence for zebra finch has 99.67% (299/300) homology to that of starlings, the target peptide sequence against which the Abcam antibody was raised is identical in both species of songbirds (Supplementary Information 1). The abcam 10275 anti-μ-OR antibody has also been used in many other species including the rodent striatum (rats: Chuhma et al., 2011; Jedynak et al., 2012; mouse: Lee et al., 2008).

### Perfusion and Histology

After behavioral recordings, birds were overdosed with ketamine and 2 mg/kg bw of xylazine and perfused transcardially with 0.01M PBS (phosphate buffered saline, pH7.4), followed by 4% paraformaldehyde containing 0.04% glutaraldehyde. Brains were cryoprotected in 30% sucrose and 40 μm thick coronal or sagittal serial cryosections were obtained for Nissl staining and immunohistochemistry (IHC).

Sequential double IHC were performed to test if μ-ORs in Area X were co-localized with DARPP-32-labeled striatal neurons (DARPP32; dopamine and cAMP-regulated phosphoprotein 32), which is present specifically in medium spiny neurons and not in other interneurons or pallidal neurons (Ouimet et al., 1998). For antigen retrieval, sections were incubated in an antigen-unmasking solution (pH 6.0; Vector laboratories, H-3300) at 80°C in a water bath (20 min), followed by quenching in 2% H_2_O_2_ (20 min). Sections were blocked with 5% Normal goat serum (NGS; Vector laboratories, S-1000) and 1% Bovine serum albumin (BSA; Vector Laboratories, A7906) for 1 h at room temperature (RT) followed by incubation in the primary anti-μ-OR antibody (1:500; made in 1% BSA and 3% NGS) at 4^∘^C for 48 h. Sections were then incubated in a secondary antibody (Alexa Fluor 488, ThermoFisher Scientific, Goat anti-rabbit, A-11008, 1:250) made in 3% NGS (3 h at RT). After rinsing, sections were incubated in 5% Normal rabbit serum (NRS, Vector Laboratories, S-5000; 1 h at RT) and then in anti-DARPP32 antibody (Abcam, ab40801, 1:500) made in 3% NGS for 12 h at 4^∘^C. Sections were then labeled with a biotinylated secondary antibody (Vector Laboratories, anti-rabbit, BA-1000, 1:250) in 3% NGS for 2 h at RT followed by incubation with Streptavidin 594 (Invitrogen, S32356, 1:200) in 3% NGS for 2 h at RT for visualization. Sections were rinsed with 0.01M PBS after every step except between blocking and primary antibody incubation. Negative controls were performed to validate the anti-μ-OR antibody (abcam 10275) by performing all steps described above but incubating sections overnight in the blocking serum instead of the primary antibody (Supplementary Information 2).

Fluorescent double immunohistochemistry was also performed on sections to study whether GABAergic neurons in Area X (visualized by staining for GAD67; glutamate decarboxylase; which is important for the decarboxylation of glutamate to GABA) express μ-ORs. Antigen retrieval was performed as mentioned above, followed by rinsing in 0.01M PBS. Sections were then incubated in 1% BSA and 5% NGS for 1 h, followed by incubation in the primary antibody solution against GAD67 (Abcam, ab26116, 1:1000) made in 1% BSA and 3% NGS at 4°C for 48 h. Double-labeling for μ-ORs was performed on the sections as given above for striatal neurons. This was followed by incubating sections in a solution containing goat anti-mouse secondary antibodies tagged with Alexa Fluor 594 (ThermoFisher Scientific, A-11005, 1:250), and Alexa Fluor 488 (ThermoFisher Scientific, Goat anti-rabbit, A-11008, 1:250) to visualize GAD67 and μ-ORs, respectively, for 3 h at RT.

A similar protocol was followed for sequential immunohistochemical staining of parvalbumin (mouse, Sigma-Aldrich, P3088, 1:500) and μ-ORs (as above) by incubating sections at 4^∘^C for 24 h. Sections were incubated in a secondary antibody tagged with Alexa Fluor 594 used for visualizing parvalbumin (ThermoFisher Scientific, Goat anti-mouse, A-11005, 1:250) and Alexa Fluor 488 (ThermoFisher Scientific, Goat anti-rabbit, A-11008, 1:250) for μ-ORs. All slides were coverslipped with a mounting medium containing DAPI (Vectashield, Vector laboratories, H-1200) and imaged using a Zeiss AxioCam MRm camera attached to a fluorescence microscope (Axioimager Z1, Carl Zeiss, Germany).

### Behavioral Analysis

Numbers of bouts, songs and introductory notes were counted following aCSF or naloxone infusion. This analysis was performed from the 6th to 35th minute of each set of recordings, to allow aCSF or naloxone to reach Area X through the tubing and microdialysis probe.

### Acoustic Properties of Motifs and Individual Syllables

The SA+ software was used to analyse raw data of spectral features [mean FM (frequency modulation), mean pitch, mean pitch goodness, mean entropy, mean AM (amplitude modulation) and mean frequency] and temporal features (syllable duration and intersyllable intervals) of songs [SA+ user manual, version 1.02 and (Khurshid et al., 2010)]. We excluded those syllable types (for example, noisy syllables) which were not present in at least three birds used for our experiments. Further, syllables marred by cage sounds (such as vocalizations of females) were excluded from our analysis. Spectro-temporal features of motifs [nine birds; *n* = 425 (aCSF); *n* = 94 (50 ng/ml naloxone); *n* = 176 (100 ng/ml naloxone)] and individual syllables in these motifs were used for comparing acoustic features before and after infusion of 100 ng/ml naloxone. The most frequently occurring syllable types in our study included (i) harmonic stacks [six birds; *n* = 435 (aCSF); 233 (naloxone)], (ii) frequency modulated syllables [eight birds; *n* = 554 (aCSF); 189 (naloxone)], (iii) high pitched [similar to pure tones; three birds, *n* = 224 (aCSF), 132 (naloxone)], and (iv) complex syllables [composed of an initial high frequency component followed by a frequency modulated component; four birds, *n* = 213 (aCSF), 136 (naloxone)]. The autocorrelation method (Kao et al., 2005) was used to measure the fundamental frequency (FF) of syllables with harmonic stacks [10 birds, aCSF (*n* = 1254 syllables); 100 ng/ml naloxone (*n* = 671 syllables); Supplementary Information 3].

### Statistics

All statistical tests were performed using Sigmaplot 13 (Systat Software, Inc.). Since the number of bouts/songs/introductory notes varied across birds during different experiments, these parameters were normalized for each bird as a percentage of the control values obtained during aCSF infusion on the same day. Normalized data was pooled and One Way ANOVA on Ranks was performed, with *P* < 0.05 deemed as significant.

Statistical tests for spectro-temporal features were performed on raw data obtained from SA+ software. Since 50 and 100 ng/ml naloxone were not always infused in the same bird, the Rank-Sum test was performed on each dataset separately and *P*-values < 0.01 were deemed significant. Similarly, levels of DA, glutamate and GABA in Area X during naloxone infusion were expressed as a percentage of the levels during aCSF infusion on the same day and One Way ANOVA on Ranks was performed for each neurotransmitter to detect the relative changes in their levels following infusion of different doses of naloxone in Area X.

## Results and Discussion

### Mu-ORs Are Expressed by Both Medium Spiny Neurons (MSNs) and Pallidal Neurons in Area X

We confirmed that μ-ORs were expressed by MSNs (Figure 1D-a) which co-express DARPP-32, a marker for striatal neurons (Figure 1D-b; Figures 1D-c and 1D-d; DAPI and merged image, respectively). The MSNs project onto pallidal neurons (Figure 1D-a) which co-express parvalbumin and μ-ORs (Khurshid et al., 2009). Earlier studies (Connor and Christie, 1999; Koehl et al., 2018) have shown that μ-ORs are inhibitory G-protein-coupled receptors and binding to their ligands (the endogenous opioids) would lead to inhibition of the neurons by which they are expressed. Blocking μ-ORs would therefore lead to the opposite effects, that is, increased excitation in these neurons. Although both MSNs and pallidal neurons express μ-ORs, pallidal neurons may be more sensitive to opioid modulation since they express higher densities of μ-ORs (Khurshid et al., 2009). Besides receiving input via MSNs, pallidal neurons also receive direct projections from HVC and in turn project to the thalamic nucleus DLM and VP, which inhibits dopaminergic VTA-SNc neurons (Gale et al., 2008). Thus, opioid modulation of pallidal neurons in Area X can potentially affect DA release as well as the acoustic properties of songs through the DLM→LMAN→ RA projection, which can influence syringeal musculature (Figure 1A).

Staining for GAD67 demonstrated a large neuron with GAD67-positive puncta present on its cell body and proximal dendrites (Figure 1D-f; labeling for μ-ORs in Figure 1D-e; DAPI in Figure 1D-g) which is typical of pallidal neurons and fainter staining associated with smaller somata in the background. The merged image (Figure 1D-h) showed that this neuron also expressed μ-ORs, as do the smaller perikarya in the background which may be striatal or other inhibitory interneurons. As demonstrated by Khurshid et al. (2009), we found that medium- and large-sized PV-positive neurons, that is, putative fast-spiking interneurons and GPi neurons (globus pallidus interna-like neurons; Goldberg et al., 2010), respectively, (Figure 1D-j; Carrillo and Doupe, 2004; Reiner et al., 2004; Yildiz and Woolley, 2017) expressed μ-ORs (Figure 1D-i; merged image, Figure 1D-l; DAPI staining in Figure 1D-k). We also found that PV-labeled neurons were more intensely positive for μ-ORs versus striatal neurons (arrowheads; Figure 1D-i; cf. Khurshid et al., 2009). Our results suggest that the effects of opioid modulation in Area X is the sum of interactions between the endogenous opioids and their ligands, not only at the level of MSNs and GPi (the two main neuronal subtypes) but also other interneurons (cf. Budzillo et al., 2017) present within this nucleus.

### Blocking μ-ORs in Area X Does Not Affect the Motivation to Sing to Females

**FIGURE 2:**
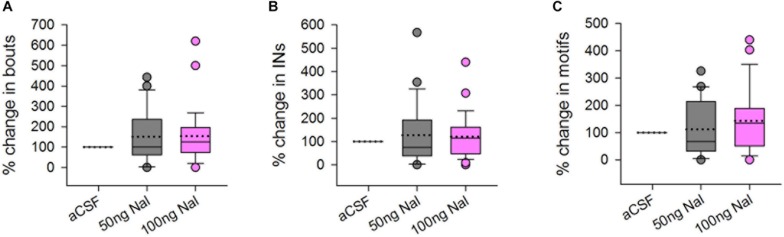
There were no significant differences in the **(A)** number of bouts, **(B)** introductory notes, or **(C)** motifs sung by male birds to females during infusions of 50 or 100 ng/ml naloxone (gray and pink boxplots, respectively) in Area X, compared to infusions of aCSF (control), represented as percentage change compared to control. Median values are represented by lines in the boxplots whereas dotted lines represent means. Circles in this figure represent outliers (5th–95th percentile) and are included in the data analysis.

There was no change in the number of FD songs, bouts or INs during naloxone infusion (50 or 100 ng/ml) in Area X compared to controls (Figures 2A–C). An earlier study showed that systemic administration of 2.5 mg/kg bw naloxone led to a decrease in FD songs whereas higher doses did not have any effect on this measure (Khurshid et al., 2010). Although site-specific infusions of naloxone in Area X cannot be directly compared to systemic administration of this antagonist, the present results suggest that song control regions other than Area X may have been involved in the decrease in FD songs observed earlier (Khurshid et al., 2010). Kelm-Nelson and Riters (2013) showed that infusing naloxone in the medial preoptic area (mPOA) of starlings (songbirds) led to increased rates of FD song in males which were poor singers, whereas the same manipulation led to lowered rates of singing in good singers, linking opioid activity in this region to FD song. Interestingly, just as in Area X, neurons in mPOA express both μ-OR and D1 receptors, suggesting that they can be modulated by endogenous opioids and dopamine in starlings (Spool et al., 2019). Other possible candidates are LMAN and VTA-SNc since they are part of the AFP, which is implicated in context-dependent singing and motivation. Yet another possibility is that μ-ORs within Area X might have a complex dose-dependent effect on the production of FD songs since the number of MSNs far exceeds that of pallidal neurons (Carrillo and Doupe, 2004) and they also differ in the levels of μ-OR expression.

### Dopamine Levels Increase Significantly in Area X During Infusions of 100 ng/ml Naloxone

**FIGURE 3:**
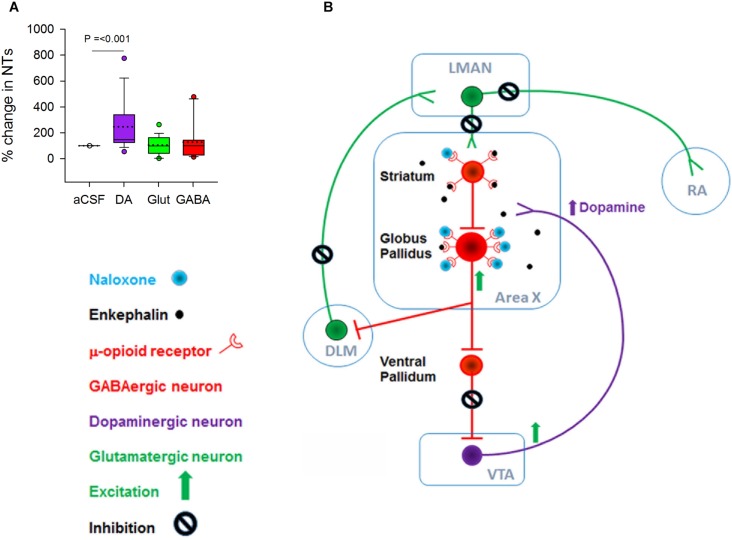
**(A)** There was a significant increase in the level of DA but not in glutamate and GABA when 100 ng/ml naloxone was infused into Area X versus control (aCSF). **(B)** Schematic showing possible mechanisms for the increase in DA during infusion of 100 ng/ml naloxone in Area X; NT, neurotransmitters. Although both striatal and pallidal neurons in Area X express μ-ORs, pallidal neurons express higher levels of these receptors, suggesting that they may be more sensitive to endogenous opioids. Blocking μ-ORs (inhibitory G-protein coupled receptors) with 100 ng/ml naloxone may disinhibit pallidal neurons. This would lead to an increase in the inhibition of ventral pallidal (VP) neurons, disinhibiting VTA-SNc neurons and ultimately leading to an increase in DA release. Additionally, disinhibition of pallidal neurons would lead to an increase in the inhibition of DLM neurons and a decrease in the activation of LMAN neurons and their targets in RA and Area X.

Although there was no change in the number of bouts, introductory notes or motifs during naloxone infusion in Area X, levels of DA increased significantly when 100 ng/ml of naloxone was infused in this region [246.52 ± 43.42(SEM) % increase, H = 15.93, d.f. = 2; *p* ≤ 0.001, Dunn’s test] (Figure 3A). Earlier studies had demonstrated a significant increase in levels of DA (Sasaki et al., 2006) and the endogenous opioid enkephalin (Wada et al., 2006) in Area X during FD song in zebra finches. The absence of significant changes in the number of FD songs in our experiments suggests that extracellular levels of DA in Area X may not be tightly linked to the number of female-directed songs that males produce (Yanagihara and Hessler, 2006; Huang and Hessler, 2008; Ihle et al., 2015), also reported by Leblois and Perkel (2012).

Why do DA levels increase in Area X during infusions of 100 ng/ml naloxone in this region? At the level of neural circuits, a decrease in the inhibition of VTA-SNc neurons by the ventral pallidum (VP) would trigger such an increase (Gale et al., 2008). Since VP neurons are inhibited by by pallidal neurons, the activation of pallidal neurons by naloxone would lead to disinhibition of VTA-SNc (Feigenbaum and Howard, 1996). A caveat is that striatal and pallidal neurons are intermixed in Area X in zebra finches (Carrillo and Doupe, 2004) and since both express μ-ORs, they would both be affected by naloxone infusions. However, our results suggest that since pallidal neurons express higher densities of μ-ORs than MSNs, they may be more sensitive to the 100 ng/ml dose of naloxone and hence more active (Figure 3B), also seen in the rodent BG (Campos-Jurado et al., 2017).

We did not observe significant changes in extracellular levels of glutamate and GABA in Area X during naloxone infusion compared to controls (Figure 3A). Previous studies (Drew et al., 2004) have demonstrated that the reuptake methods for both GABA and glutamate at the synaptic junctions (Watson et al., 2006) are very efficient, thus preventing leakage of significant levels of either of these neurotransmitters into the extracellular fluid. It is possible that the lack of significant changes in the levels of GABA and glutamate during naloxone infusion in Area X may have occurred as a result of these mechanisms.

### Acoustic Features of Motifs and Individual Syllables Are Altered During Naloxone Infusion in Area X

**FIGURE 4:**
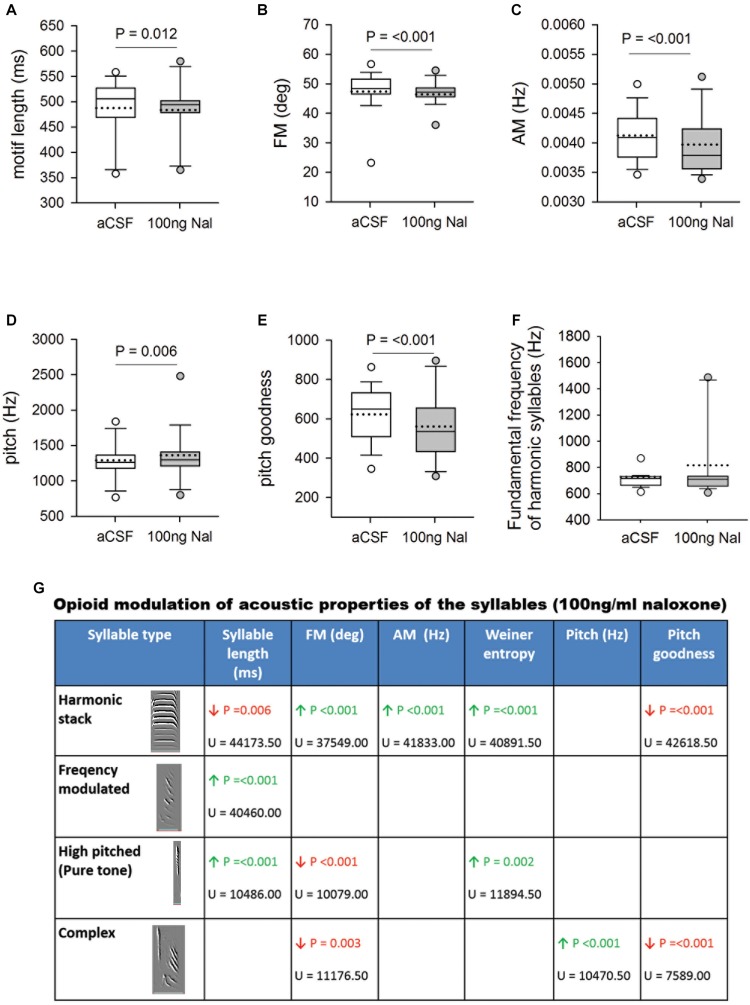
**(A)** Blocking μ-ORs with 100 ng/ml naloxone in Area X led to a significant decrease in motif length versus controls. Significant changes in spectral features following infusions of 100 ng/ml naloxone in Area X led to decreases in **(B)** FM, and **(C)** AM, increase in **(D)** pitch and a decrease in **(E)** pitch goodness of motifs versus aCSF infusions in Area X. **(F)** The fundamental frequency of harmonic syllables was not affected by blocking μ-ORs in Area X. **(G)** Summary of changes in the acoustic features of different syllables in FD songs during infusions of 100 ng/ml naloxone in Area X versus control. Empty cells imply no change, green arrow represents significant increases and red arrows represent significant decreases with *P*-value set to <0.01. The U-statistic (Mann–Whitney Rank Sum Test) is mentioned below *P*-values.

Whereas infusing 50 ng/ml naloxone in Area X had no effect, acoustic features of motifs and individual syllables were altered by infusions of 100 ng/ml of naloxone in this region (Figure 4). Changes included significant decreases in motif length (aCSF: 487.67 ± 3.07 versus Naloxone: 483.77 ± 4.51, *U* = 32534.50), FM (47.40 ± 0.38 versus 46.45 ± 0.51, U = 28942.50), AM (0.00413 ± 0.000022 versus 0.00397 ± 0.000041, *U* = 26991) and pitch goodness (622.56 ± 7.31 versus 561.04 ± 13.23, *U* = 28109), whereas the pitch of motifs increased significantly (1292.53 ± 15.67 versus 1363.68 ± 29.72, *U* = 32057; Figures 4A–E) compared to controls. Similar results (decreases in FM, AM, and pitch goodness) of FD motifs had been reported earlier (Khurshid et al., 2010), following systemic administration of naloxone in male zebra finches. Of the different syllable types in the songs of zebra finches, the fundamental frequency (FF) of syllables structured as harmonic stacks are known to be more variable in undirected song compared to FD song (Kao et al., 2005). Further, Leblois et al. (2010) demonstrated that these social-context mediated changes in FF of harmonic stacks are abolished if the D1-subtype of DA receptors is blocked in Area X. In our study, we found that FF of harmonic stacks in FD song did not change significantly during naloxone versus aCSF infusions in Area X, despite the increase in DA levels (Figure 4F). We also found that alterations in the acoustic features of motifs (cf. Khurshid et al., 2010) could be attributed to those in two or more of the commonly occurring syllable types in our birds (harmonic stacks, high-pitched, frequency-modulated and complex; Figure 4G). Finally, we did not find any changes in the coefficient of variation (CV) of either motifs or individual syllables for any of the spectro-temporal features (including fundamental frequency), suggesting that blocking μ-ORs in Area X did not increase variability in songs.

To test whether there were significant variations in FD song across different experimental days, we compared each acoustic feature for all motifs sung during aCSF or naloxone infusion in Area X during the four experimental days. There were no significant differences in motif length, FM, AM, pitch, and pitch goodness of motifs within each group (Supplementary Information 4). These results suggest that alterations in these acoustic features are consistent across every day of the experiment. Further, the effects of infusing naloxone to block μ-ORs in Area X do not persist until the next experimental day.

How does blocking μ-ORs in Area X lead to changes in the spectro-temporal properties of songs? We speculate that GPi activity would increase as a result of blocking μ-ORs expressed by these neurons since they are inhibitory in nature and also as a result of increased DA levels in Area X (cf. Woolley and Kao, 2015). In both cases, DLM neurons would be inhibited, further inhibiting LMAN whose activity is important for driving variability in song (Kao et al., 2005; Kao and Brainard, 2006), which is consistent with our results.

Further, the spectral structure of syllables is controlled by the vocal motor nucleus RA, which controls syringeal and respiratory musculature through its downstream projections to the hypoglossal nucleus (Vicario, 1991). In the present study, perturbing opioid modulation in Area X may have affected RA in three ways. Firstly, the increase in DA during infusions of 100 ng/ml naloxone in Area X that we observed may have contributed to changes in the spectral properties of FD song via the Area X→ DLM→LMAN→ RA pathway. Leblois et al. (2010) have demonstrated that increases in DA in Area X via D1 receptors increase intrinsic activity of GPi neurons and reduce the variability in their firing. Increasing DA levels in Area X led to a decreased response in LMAN via DLM to playbacks of BOS and electrical stimulation of HVC (Leblois et al., 2010). These results suggest that the increase in DA during infusions of 100 ng/ml naloxone in Area X in our study may have contributed to changes in the spectral properties of FD song via LMAN and its projections to RA.

Secondly, it is possible that excitatory DLM neurons which receive input from Area X and project to LMAN would be inhibited by the activation of GPi neurons following naloxone infusions. A decrease in DLM excitation would lead to decreased neural activity in LMAN, which in turn, could alter neuronal activity in RA. Taken together, these results suggest a synergistic action of DA and the opioid system on the activity of GPi output and the modulation of acoustic features of FD song (Figure 3). Thirdly, VTA (A10) provides dopaminergic input to RA (Appeltants et al., 2002) and alterations in the activity of this pathway as a result of changes in GPi which is upstream may have modulated the spectral features of song directly.

In contrast to spectral features, the song control nucleus HVC codes for the timing and sequence of individual syllables in the motifs and projects to RA (Leonardo and Fee, 2005). Perturbations generated in Area X by blocking μ-ORs may have reached HVC via the Area X→ VP→ A11→ HVC or alternatively, via the Area X→ VP→ VTA→ HVC circuit; (Appeltants et al., 2000). However, it is unlikely that altered DA levels in Area X in our study affected the timing of FD songs since D1R modulation in this region did not affect the temporal structure of syllables (Leblois and Perkel, 2012).

Interestingly, changes in acoustic parameters did not occur uniformly in different syllable types while μ-ORs were blocked in Area X. For example, the only difference before and during treatment in FM notes was a significant increase in syllable length. However, other syllable types (HS and HP) underwent significant changes in various spectral features besides length (Figure 4G). Similar changes in the acoustic features of individual syllables and motifs were observed following consumption of alcohol in zebra finches, which varied depending on the syllable type (Olson et al., 2014). These findings suggest that neural circuitry and/or syringeal musculature required to produce various syllable types may be different. Further, each set of circuits underlying a particular syllable type may be more or less sensitive to opioid modulation, depending on the number of ORs associated with each. Our results also suggest that perturbations in BG functions as a result of blocking or stimulating ORs may alter vocalizations, such as those in addicts.

## Summary and Conclusion

Whereas blocking μ-ORs in Area X did not affect the motivation of male zebra finches to sing FD song, changes in spectro-temporal features of motifs and individual syllables and an increase in the levels of DA in Area X were triggered by infusing 100 ng/ml of naloxone in Area X. Our results suggest that μ-ORs modulate vocalization and may be partly responsible for the vocal impairments observed in drug-addicts and PD patients (Prosser et al., 2006; Rusz et al., 2015). Besides changes in spectral features of zebra finch songs as a result of increased DA in Area X (Leblois et al., 2010; Leblois and Perkel, 2012), lesions in Area X created using 6-OHDA (6-hydroxydopamine; a neurotoxin which selectively destroys DA neurons and synapses; Miller et al., 2015) also led to changes in the vocalizations of zebra finches. In such a case, it is possible that the unopposed action of endogenous opioids following FD song would further inhibit striatal neurons. Interestingly, blocking opioid receptors in the basal ganglia have been shown to alleviate the symptoms of Levodopa-induced dyskinesia (LID; Pan and Cai, 2017). These findings are thought to result from lowering the aberrant activity of MSNs which express both μ-ORs and DA receptors (Ryan et al., 2018). Taken together with these findings, our results suggest that the μ-OR and dopaminergic systems interact with each other to modulate the output of the basal ganglia. Given the similarities in the underlying neural circuits in humans and songbirds, zebra finches are an excellent model system to study the interactions of various neuromodulators and their effects on learned vocalizations.

In future, these questions could be addressed with cell-type specific knockdowns of μ-OR/D1R/D2R and/or optogenetic studies which would give more insight into the interplay between μ-ORs and DA within Area X. Songbirds may, therefore, be used as model systems to further explore how opioid neuromodulation within the BG controls vocalization and speech production.

## Data Availability

The datasets generated for this study are available on request to the corresponding author.

## Ethics Statement

This study was carried out in accordance with the recommendations of the Committee for the Purpose of Control and Supervision of Experiments on Animals (CPCSEA), India. The protocol was approved by the Institutional Animal Ethics Committee at the National Brain Research Centre, Manesar, India.

## Author Contributions

SK and SI designed the experiments, performed the statistics, interpreted the data, and drafted the manuscript. SK and AM performed the surgeries and behavioral experiments. SK, AM, and RR analyzed the behavioral data. SK and HS designed and performed the LC-MS experiments. HS and TV analyzed the LC-MS data. US helped in surgeries and standardized the anesthesia. NK helped in performing the basic electrophysiology during surgeries. SI conceived and supervised the study. All authors contributed to the scientific discussions and approved the final manuscript.

## Conflict of Interest Statement

The authors declare that the research was conducted in the absence of any commercial or financial relationships that could be construed as a potential conflict of interest.
