# Supplementary figures and images for: Altering Opioid Neuromodulation in the Songbird Basal Ganglia Modulates Vocalizations
Source: Front Neurosci. 2019 Jul 3;13:671. doi: 10.3389/fnins.2019.00671 (PMC6618663; doi:10.3389/fnins.2019.00671)

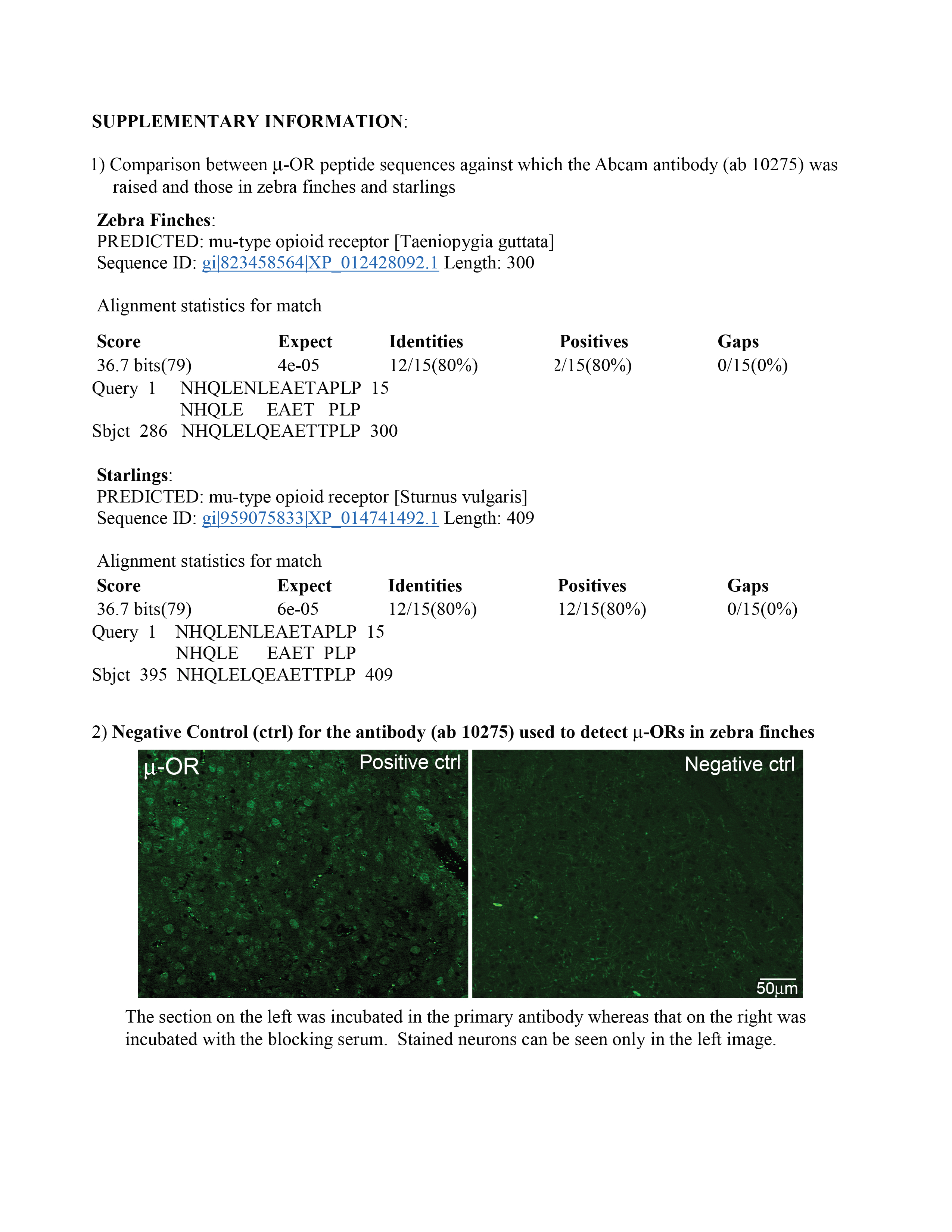

Supplement: Supplementary file 1 [file Image_1.tiff]
